# Pharmacological treatment and vaccines in monkeypox virus: a narrative review and bibliometric analysis

**DOI:** 10.3389/fphar.2023.1149909

**Published:** 2023-05-05

**Authors:** Muhammad Aaqib Shamim, Prakisini Satapathy, Bijaya Kumar Padhi, Sai Dutt Veeramachaneni, Naushaba Akhtar, Anindita Pradhan, Abhimanyu Agrawal, Pradeep Dwivedi, Aroop Mohanty, Keerti Bhusan Pradhan, Russell Kabir, Ali A. Rabaan, Jawaher Alotaibi, Zainab A. Al Ismail, Zainab Ahmed Alsoliabi, Ali Al Fraij, Ranjit Sah, Alfonso J. Rodriguez-Morales

**Affiliations:** ^1^ Department of Pharmacology, All India Institute of Medical Sciences, Jodhpur, India; ^2^ Department of Virology, Postgraduate Institute of Medical Education and Research, Chandigarh, India; ^3^ Department of Community Medicine, School of Public Health, Postgraduate Institute of Medical Education and Research, Chandigarh, India; ^4^ PSG College of Pharmacy, Coimbatore, India; ^5^ Indian Council of Medical Research—Regional Medical Research Centre, Bhubaneswar, India; ^6^ Department of Pharmacology, All India Institute of Medical Sciences, Bathinda, India; ^7^ Centre of Excellence for Tribal Health, All India Institute of Medical Sciences, Jodhpur, India; ^8^ All India Institute of Medical Sciences, Gorakhpur, India; ^9^ Department of Healthcare Management, Chitkara University, Chandigarh, India; ^10^ School of Allied Health, Anglia Ruskin University, Essex, United Kingdom; ^11^ Molecular Diagnostic Laboratory, Johns Hopkins Aramco Healthcare, Dhahran, Saudi Arabia; ^12^ Infectious Diseases Unit, King Faisal Specialist Hospital and Research Center, Department of Medicine, Riyadh, Saudi Arabia; ^13^ Long Term Care Department, Dhahran Long Term Hospital, Dhahran, Saudi Arabia; ^14^ Pharmacy Department, Qatif Central Hospital, Qatif, Saudi Arabia; ^15^ Medical Laboratories and Blood Bank Department, Jubail Health Network, Jubail, Saudi Arabia; ^16^ Tribhuvan University Teaching Hospital, Institute of Medicine, Kathmandu, Nepal; ^17^ Harvard Medical School, Boston, MA, United States; ^18^ Dr. D. Y. Patil Medical College, Hospital and Research Centre, Dr D. Y. Patil Vidyapeeth, Pune, Maharashtra, India; ^19^ Faculty of Health Sciences, Universidad Científica del Sur, Lima, Peru; ^20^ Gilbert and Rose-Marie Chagoury School of Medicine, Lebanese American University, Beirut, Lebanon

**Keywords:** mpox infection, antiviral, drug, management, public health emergency, tecovirimat, cidofovir, bibliometry

## Abstract

Mpox (earlier known as monkeypox) virus infection is a recognized public health emergency. There has been little research on the treatment options. This article reviews the specific drugs used to treat mpox virus infection and the vaccines used here. Instead of focusing on the mechanistic basis, this review narrates the practical, real-life experiences of individual patients of mpox virus disease being administered these medicines. We conducted a bibliometric analysis on the treatment of the mpox virus using data from several databases like PubMed, Scopus, and Embase. The research on this topic has grown tremendously recently but it is highly concentrated in a few countries. Cidofovir is the most studied drug. This is because it is indicated and also used off-label for several conditions. The drugs used for mpox virus infection include tecovirimat, cidofovir, brincidofovir, vaccinia immune globulin, and trifluridine. Tecovirimat is used most frequently. It is a promising option in progressive mpox disease in terms of both efficacy and safety. Brincidofovir has been associated with treatment discontinuation due to elevated hepatic enzymes. Cidofovir is also not the preferred drug, often used because of the unavailability of tecovirimat. Trifluridine is used topically as an add-on agent along with tecovirimat for ocular manifestations of mpox virus disease. No study reports individual patient data for vaccinia immune globulin. Though no vaccine is currently approved for mpox virus infection, ACAM 2000 and JYNNEOS are the vaccines being mainly considered. ACAM 2000 is capable of replicating and may cause severe adverse reactions. It is used when JYNNEOS is contraindicated. Several drugs and vaccines are under development and have been discussed alongside pragmatic aspects of mpox virus treatment and prevention. Further studies can provide more insight into the safety and efficacy of Tecovirimat in actively progressing mpox virus disease.

## 1 Introduction

While the world is finding ways to deal with SARS-CoV-2 and COVID-19, a novel threat of mpox has emerged in the human population. The World Health Organization (WHO) has declared this a health emergency, and the confirmed cases have risen to 84,916 and 81 deaths as of 20 January 2023. One hundred ten countries throughout the globe have reported cases (World Health Organization, 2022a). The mpox virus is a member of the orthopoxvirus family. It generally invades rodents and animals but has now escaped into the human population. There are two distinct genetic clades of MPXV: African clad (Congo basin) and West Africa clad (Kaler et al., 2022). As per reports by WHO, new cases have been identified from various regions of the world, irrespective of their historical distribution (Reynolds et al., 2017). However, the symptoms are diverse and less severe than in smallpox (Rizk et al., 2022; Satapathy et al., 2022; Gandhi P et al., 2023).

Treatment of the mpox virus is a new challenge for the entire healthcare system (Sherwat et al., 2022). There are very few studies on it, and there is an urgent need to address it. There have been a few review articles covering the treatment options being employed. However, given that mpox virus disease is a rapidly evolving field, we regularly find new original research articles cropping up. We found several original research articles reporting on treatment options and other aspects of mpox virus disease that were not discussed in previous review articles (DeLaurentis et al., 2022; McCarthy, 2022; Torres, 2022; Rabaan et al., 2023; Shamim et al., 2023). This paper gives a brief analysis of literature from the past till the current time about the pharmacological treatment of the disease and the drugs being administered along with the vaccines being used. This review gives a detailed insight into the clinical orientation of the antivirals being administered: tecovirimat (TPOXX), cidofovir, brincidofovir, trifluridine, and Vaccinia Immune Globulin which would be essential for improving the treatment protocol and for increasing the treatment efficacy of the disease (Siegrist and Sassine, 2022). We follow it up with a discussion on the new and upcoming options for both the treatment and prevention of mpox virus disease.

## 2 Bibliometric analysis

The bibliometric analysis involves using mathematical methods to study books and communication media (Smith, 2008). It is beneficial in assessing the trend of research on a specific topic. This can help identify gaps in the currently available literature and thus propose ideas for future research. The bibliometric analysis covered three electronic databases for all articles from the inception of each database till 22 December 2022. The keywords “monkeypox” and “mpox” were used in combination with “treatment”/“management”/“drug” in the title, abstract, and keywords in Scopus, PubMed, and Embase, yielding 722, 370, and 289 results, respectively (Table 1). The distribution of studies depending on the year of publication and type of studies is shown in (Table 2). Only the last 5 years have been mentioned for clarity. A greatly increased number of studies can be noticed in 2022 (compared to previous years) owing to the public health emergency. This pattern highlights the relevance of research in this vastly underexplored area of public health concern.

**TABLE 1:** The adjusted search terms as per searched electronic databases.

Database	No	Search query	Results
PubMed			
	#1	{[monkeypox (Title/Abstract)] OR [mpox (Title/Abstract)]} OR [mpxv (Title/Abstract)]	2,262
	#2	{[treatment (Title/Abstract)] OR [management (Title/Abstract)]} OR [drug(Title/Abstract)]	6,827,326
	#3	#1 AND #2	370
Scopus			
	#1	[TITLE-ABS-KEY (monkeypox) OR TITLE-ABS-KEY (mpox)]	2,523
	#2	{[TITLE-ABS-KEY (treatment) OR TITLE-ABS-KEY (management) OR TITLE-ABS-KEY (drug)]}	19,496,403
	#3	#1 AND #2	722
Embase			
	#1	[(monkeypox:ti,ab) OR (mpxv:ti,ab)] OR (mpox:ti,ab)	1,734
	#2	[(treatment:ti,ab) OR (management:ti,ab)] OR (drug:ti,ab)	7,832,676
	#3	#1 AND #2	289

**TABLE 2:** Results of the bibliometric analysis of the search for “Monkeypox” in title, abstract, and keywords in PubMed, Scopus, and Embase (on 18 December 2022).

Type (Scopus)	Frequency	Year	Scopus	PubMed	Embase
Article	395	2022	309	243	147
Review	201	2021	21	7	5
Letter	38	2020	19	11	6
Note	38	2019	15	5	6
Miscellaneous	50	2018	12	3	6

^a^ Refers to the total number of a specific type of publication from 2018 to 2022.

If we further look at the countries where research is ongoing in the field of mpox virus disease, we can notice a worrying trend (Table 3). Though the United States expectedly has the highest number of studies, there are very few studies from other countries. As an international issue concerning most countries across the globe, we need efforts from all countries to help combat this situation. Globally, efforts should be taken to promote equitable research efforts.

**TABLE 3:** Results of the bibliometric analysis with regard to country of publication as per Scopus.

Country	Frequency
United States	347
United Kingdom	61
India	51
China	36
Germany	36
Belgium	24
France	24
Italy	24
Canada	22
Russian Federation	20

There is a clear trend in the funding patterns in research pertaining to the mpox virus (Table 4). The National Institute of Allergy and Infectious Diseases funds the highest number of studies on this public health emergency. Most (80%) of the top ten funding agencies are from the United States. The other sponsors (one each) are from China and United Kingdom.

**TABLE 4:** Results of the bibliometric analysis with regard to funding sponsor of publications as per Scopus.

Rank	Funding agency	Country	Publication count
1	National Institute of Allergy and Infectious Diseases	United States	106
2	National Institutes of Health	United States	51
3	National Center for Research Resources	United States	14
4	National Natural Science Foundation of China	China	12
5	Centers for Disease Control and Prevention	United States	11
6	National Cancer Institute	United States	11
7	Defense Threat Reduction Agency	United States	10
8	United States Department of Health and Human Services	United States	8
9	Biomedical Advanced Research and Development Authority	United States	7
10	Medical Research Council	United Kingdom	7

**FIGURE 1:**
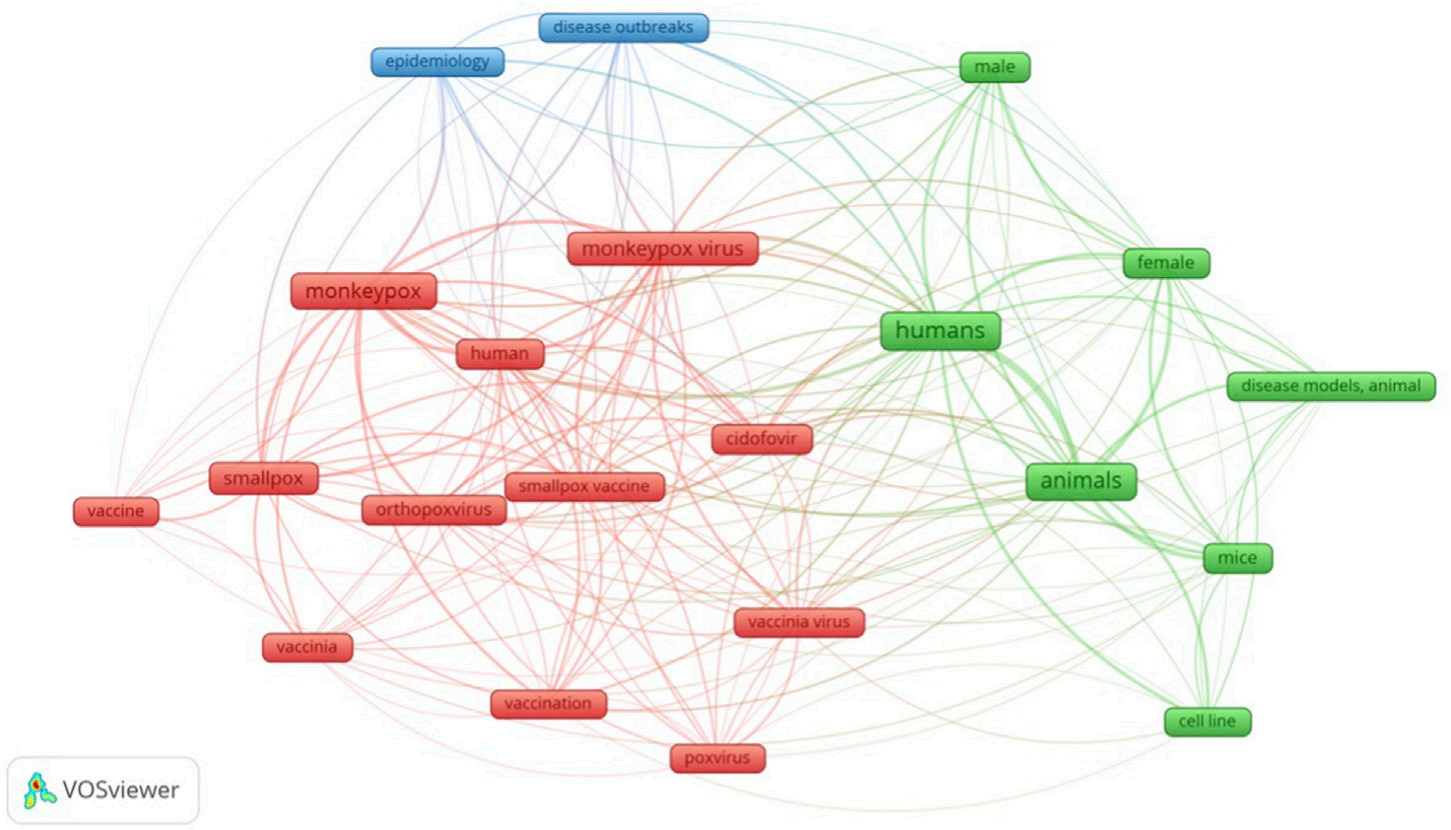
Visual network of keywords in published literature pertaining to treatment of monkeypox infection.

Using the results of the aforementioned search strategy, a bibliometric map of the relevant keywords was constructed using VOSviewer. We performed a co-occurrence analysis of keywords across several databases using whole counting. This helped us visualize the critical areas discussed in the sparsely available literature on the treatment of mpox. Figure 1 illustrates this.

There is a strong co-occurrence of “humans” and “animals” keywords. Many of the studies on pharmacological management of mpox virus infection have been based on animal models, and human studies have only recently started cropping up. Cidofovir is mentioned a lot. This is because it is used in many other conditions apart from mpox virus infection, more than any other antiviral mentioned here. It is mainly used against cytomegalovirus. It is used in patients with acquired immunodeficiency syndrome (AIDS) and those undergoing organ transplantation. It also demonstrates *in-vitro* activity against several DNA viruses like herpesvirus, poxvirus, polyomavirus, and papillomavirus (de Clercq, 2003). It is used off-label in respiratory papillomatosis (Ballestas et al., 2021), acyclovir-resistant herpes simplex virus (Enescu et al., 2021), skin lesions associated with herpesvirus (Ferrer et al., 2021), BK polyomavirus (Napolitano et al., 2021). We have thus built upon the existing bibliometric analyses pertaining to mpox virus disease (Cheng et al., 2022; Zeeshan et al., 2022). We have focused on the treatment aspect for our bibliometric analysis.

## 3 Treatments and vaccines

### 3.1 Tecovirimat (alternative name: ST-246, brand name: TPOXX^®^)

Tecovirimat is the most commonly employed antiviral in patients with mpox infection. Initially identified in a high throughput screening, the United States FDA granted it approval in 2018, and it is indicated in smallpox disease (Grosenbach et al., 2011). Tecovirimat was approved under the FDA animal rule after testing on monkeys and rabbits, and an investigational new drug protocol has been granted to the United States Centers for Disease Control to study it in non-variola orthopoxvirus infections, including mpox virus (Food and Drug Administration, 2018; Centers for Disease Control and Prevention, 2022b). Due to concerns pertaining to bioterrorism, the United States has stockpiled around two million doses to TPOXX and other drugs acting against mpox (Hoy, 2018).

The mpox virus enters the cytoplasm of human cells. After pre-processing, it replicates its DNA in Guarnieri inclusion bodies (Pauli et al., 2010). Then, the synthesised protein particles are assembled to form an intracellular virion. This undergoes maturation followed by enveloping. This prepares the mature virion to now exit the cell. The membranes of the virion and the cell fuse, and the extracellular mature virion exits the cell. It goes on to infect other human cells, thus amplifying the disease. The VP37 protein of the mpox virus is responsible for this enveloping of mature extracellular virions. Golgi-derived membrane is used for this envelopment. Tecovirimat inhibits this VP37 protein. Thereby, this enveloping of the mature viral particle is inhibited even though DNA synthesis and viral maturation progresses normally. The absence of enveloping ensures that the matured virion cannot properly exit the infected cells, systemically spread in the body, and infect other cells. Thus, TPOXX inhibits the propagation of mpox virus disease to other human cells and hinders disease progression (Hudu et al., 2023). VP37 also interacts with human proteins like TIP47 and Rab9. TPOXX inhibits this, further diminishing propagation of mpox virus in the human body (Grosenbach et al., 2011). Cross-resistance to cidofovir or brincidofovir is not expected (Duraffour et al., 2007).

TPOXX has several stereoisomeric forms. The monohydrate form has low solubility but considerable permeability. Amongst the several polymorphic forms available, the first form of Tecovirimat monohydrate crystal is packed in capsules after recrystallizing using water and ethyl acetate. This is preferred due to the higher thermodynamic stability compared to other forms (European Medicines Agency, 2021). It is usually administered as capsules containing 200 mg of the active drug. But for those weighing less than 13 kg, the intravenous formulation is administered as a slow infusion. One crucial difference between the two dosage forms is that only the latter is contraindicated in patients with renal disease (creatinine clearance of 30 mL/min or less). Even in cases with mild-to-moderate renal impairment (creatinine clearance ranging from 30 to 89 mL/min) or in the pediatric population aged less than 2 years, caution is required. Thus, clinicians should routinely test this in all patients before starting Tecovirimat infusion. The reason behind this selective toxicity is the presence of an excipient hydroxypropyl-β-cyclodextrin in the parenteral preparation. This excipient is added due to the poor water solubility of Tecovirimat. Caution is needed in comorbid or multimorbid patients, especially in those with diabetes or history of seizures. It is also known to have drug-drug interactions with midazolam and repaglinide. When co-administered with Repaglinide or Midazolam, monitoring for hypoglycemia or Midazolam effectiveness is recommended. Drugs metabolized by CYP3A and CYP2B6 may show diminished therapeutic activity due to induction of these hepatic enzymes. A synergistic drug-drug interaction has also been reported. An *in silico* study has shown better efficacy against mpox virus with combination of atovaquone and TPOXX (Akazawa et al., 2022). Some of the common associated adverse events are headaches, gastrointestinal disturbances, and injection site complaints like pain, swelling, erythema and extravasation (De Clercq et al., 2022; Food and Drug Administration, 2022; Siegrist and Sassine, 2022). Amongst food-drug interactions, administration of this drug is with milk or yogurt is recommended. Though teratogenicity was observed in mice, but the results have not been replicated in rabbits. Thus, future studies should consider this gap in literature. It might be less effective in immunocompromised individuals. Amongst other pharmacokinetic parameters, it has a half-life of four to 6 h and a volume of distribution of 1,030 L following an oral dose of 600 mg. 77%–82% of the drug is protein-bound. Elimination is primarily by hepatic conjugation followed by renal excretion. Some portion is also excreted unchanged via faeces. It has a clearance of 31 L per hour (European Medicines Agency, 2021; Food and Drug Administration, 2022).

There are several studies on the use of Tecovirimat in monkeypox disease in humans. It has been effective in arresting the aggravation of the disease. A female in her 30 s developed mpox virus lesions over her thorax. On testing positive, she was started on Tecovirimat. New lesions stopped cropping up within a day, and lesions turned PCR negative within 2 days (Adler et al., 2022). A man with an extensive facial pustular lesion was administered Tecovirimat and improved (Rao et al., 2022). Another male patient developed genital lesions that gradually spread throughout the body. After starting Tecovirimat, the development of new lesions stopped within 2 days, and the patient recovered. In an HIV-positive patient, oedema over palatine tonsil and pain while ingesting food benefitted from Tecovirimat. Another patient did not improve with initial empirical treatment. With Tecovirimat, lesions started crusting and improved by the second day itself (Matias et al., 2022). Progressive oral symptoms not responding to several lines of treatments started improving within 2 days of initiating Tecovirimat (Ajmera et al., 2022). In this uncontrolled cohort study, with 25 participants receiving Tecovirimat, 23 recovered as per the reported 21-day outcome. In one individual, there were no new lesions. However, in another patient, new lesions were still developing despite having received a 21-day course of Tecovirimat, unlike the 14-day course prescribed to others (Desai et al., 2022). A report describes two cases of severe proctitis. Both had lesions spread throughout the body. However, the rectal lesions were very prominent and caused enough pain to require opioids. In both cases, rapid improvement was seen within 36–48 h (Lucar et al., 2022). Another male patient complaining of proctitis recovered with a course of TPOXX after empirical treatment with doxycycline, valacylovir, and benzathine penicillin G failed (37065384).

A patient presented with ulcerative lesions over the tip of the tongue and over the anterior aspect of its ventral surface. Lesions later spread throughout his body. Though he is still symptomatic, as per the last update, he has been improving with Tecovirimat (Peters et al., 2022). An attendee of a pride festival reported macules, papules, and pustules across the body. He also tested positive for Herpes Simplex Virus—2 alongside the mpox virus. He was co-treated with Tecovirimat and Valacyclovir (for Herpes Simplex Virus—2). He continued developing new lesions and was febrile for the first 2–3 days. He was then discharged as he turned afebrile and improved (Shaw et al., 2022). An uncontrolled cohort study in the Democratic Republic of Congo reports 14 patients with monkeypox virus infection and treated with Tecovirimat. Patients started showing signs of improvement, like suppression of active lesions from day 2 of Tecovirimat administration. All patients were better by the end of 2 weeks. Some lesions and lymphadenopathy persisted, but all 14 participants improved symptomatically (Mbrenga et al., 2022). This patient with multiple comorbidities like syphilis, Kaposi sarcoma, HIV, and hypertension reported papules, vesicles, and pustules throughout the body. The patient was started on Tecovirimat, and he improved rapidly (Hernandez et al., 2022). Two patients with mpox virus infection had encephalomyelitis. They presented with progressive hemiparesis, paraparesis, bowel and urinary abnormalities, and altered mentation, among other manifestations. Tecovirimat was administered orally to both patients. They improved and were subsequently discharged (Pastula et al., 2022). A 35-year-old female with encephalitis and transverse myelitis. Empirical acyclovir was stopped, and tecovirimat was initiated after the patient turned out to be positive for the mpox virus. The neurological pathology continued to progress, and she was started on methylprednisolone and cidofovir in the fourth week of the illness. She gradually improved. This could be attributed to the synergistic action of tecovirimat and cidofovir (Cole et al., 2022). A patient with progressive generalized mpox virus dermatological lesions and watery diarrhea was started on Tecovirimat. All the issues started improving within two to 3 days (Viguier et al., 2022). This case series reports three participants with various comorbidities like ulcerative colitis and syphilis. Treatment was initiated with Tecovirimat. In two of the patients, this led to fading away of existing lesions, no appearance of newer lesions, along with an immediate benefit seen clinically. In the third patient, clinical response was seen but was slow. However, C-reactive protein and viral DNA load were reduced in the first week itself (Nörz et al., 2022). This study reports twenty participants who received tecovirimat. All the patients recovered. Participants reported improvement within two to 3 days (Wu et al., 2022). Two patients with concomitant HIV and anogenital and rectal mpox virus infection recovered with tecovirimat (Beatty et al., 2022). A case with disseminated ocular involvement was administered tecovirimat (Rimmer et al., 2023). All fifteen patients started on tecovirimat improved, and no new lesions developed (Mondi et al., 2022). This 35-year-old man with multiple swollen facial pustules. He was started on tecovirimat and improved (Manoharan et al., 2022). This patient presented with multiple lesions throughout the body, including eyes. All the lesions improved with tecovirimat (Rai et al., 2022). This study reports on the usage of tecovirimat under an investigational new drug protocol. 230 of 317 patients recovered, while most of the remaining 87 had not yet completed the 14-day course of tecovirimat (O’Laughlin et al., 2022). A recent case report on a patient with HIV not responding adequately to TPOXX have given rise to concerns regarding emerging resistance to TPOXX and the need to consider immune reconstitution inflammatory syndrome in such cases (36992234).

It has been associated with serious adverse events only in one study conducted in the Democratic Republic of Congo. Here, one patient developed severe anemia, with his hematocrit dropping to as low as 18%. He later recovered. Another patient was discharged after 2 weeks of treatment as his lesions were improving, and he turned out PCR negative. However, he died 3 days after that. In both cases, they considered the event unrelated to or unlikely to be related to this drug (Mbrenga et al., 2022). Some other studies have reported a few adverse events too. One patient reported loose stool after every dose. Another developed elevated alanine aminotransferase on the sixth day of treatment. This reduced over the next 2 days returning back to normal values without discontinuation of treatment. The derangement of hepatic enzyme resolved independently (Matias et al., 2022). Two more patients developed a transient rise in hepatic enzymes. Alanine aminotransferase and aspartate aminotransferase were elevated to 97 IU/L and 86 IU/L, respectively. This resolved on its own (Viguier et al., 2022). Another patient was quite different because he developed elevated gamma-glutamyl transferase levels of 277 U/L. However, his transaminases remained within range. Despite this different pattern of hepatic enzyme derangement, this was also only a transient rise (Nörz et al., 2022). Fatigue, headache, backache, and nausea are some of the other reported adverse events (Desai et al., 2022; O’Laughlin et al., 2022; Wu et al., 2022). Summing up, Tecovirimat is a promising option in terms of both efficacy and safety in worsening mpox virus disease.

Two clinical trials are going on to evaluate the efficacy and safety of Tecovirimat in mpox virus infection (NCT05534984, NCT05534165). The multinational randomized controlled trial has started recruiting. The second trial, which is based in Canada, is yet to start recruiting. Both are expected to be completed by the following year, i.e., 2023 (Ortiz-Saavedra et al., 2022).

### 3.2 Cidofovir (brand name: Vistide^®^)

Cidofovir is used in diseases due to cytomegalovirus (CMV), herpesviruses, and several DNA viruses. It is indicated in certain CMV diseases in immunocompromised people (Cherrington et al., 1998; Kendle and Fan-Havard, 1998) and has been used off-label in several conditions caused due to DNA viruses (Razonable, 2011).

Cidofovir is a nucleoside analogue. Using intracellular metabolism, it is activated into cidofovir-diphosphate, which competitively inhibits viral DNA polymerase, thereby interfering with viral DNA synthesis. It inserts into the viral genomic material, inhibiting further prolongation (Lea and Bryson, 1996). Unlike substances acting on A48R, cidofovir inhibits human DNA polymerases also. However, its activity here is eight to six hundred times less than for the viral enzyme (https://pubmed.ncbi.nlm.nih.gov/30397065/). Pharmacologically, it is an example of a hit-and-run drug. Administered intravenously, the serum concentration of the drug falls rapidly following the infusion, and it has a short plasma half-life of 2 h. However, the intracellular half-life of the active form is as high as 65 h. Cidofovir undergoes renal elimination, and this involves a critical drug-drug interaction. Probenecid blocks tubular secretion of this drug, thereby reducing its excretion and increasing its serum level (Cundy, 1999; Wolf et al., 2003). Nephrotoxicity is a common clinical concern with this drug, and hydration and probenecid are recommended to reduce its incidence (Lea and Bryson, 1996; Kazory et al., 2007). Relevant monitoring is recommended during therapy due to the risk of ocular complications (like hypotony, uveitis, and iritis) and myelosuppression (Ambati et al., 1999; Tseng et al., 1999).

In treating mpox, cidofovir has been used clinically in at least four reports. In three of the cases, it was only due to the unavailability of tecovirimat (Mailhe et al., 2022; Moschese et al., 2022; Raccagni et al., 2022). One patient developed vesicles over his nose along with suspected bacterial superinfection. He improved with cidofovir and antibiotics (Moschese et al., 2022). In this case of atypical presentation of mpox with ophthalmic manifestations like the involvement of cornea, conjunctiva, and eyelids, cidofovir was administered. However, the report says that the lesions are still evolving in spite of two intravenous injections (these are administered weekly (Mailhe et al., 2022). Another similar ophthalmic presentation of mpox was administered cidofovir. However, he reported improvement in 3 days (Scandale et al., 2022). Raccagni et al. (2022) describe four patients in Italy using cidofovir. They had varying clinical presentations ranging from pharyngeal and ocular involvement to rectal and genital involvement. They were given single-dose cidofovir along with hydration and probenecid, and they improved. In this cohort study, 12 patients were administered add-on topical cidofovir while others were on standard care. Topical cidofovir was associated with quicker clearance of lesions and higher resolution of lesions as per PCR testing (Sobral-Costas et al., 2022). A patient with co-infection with HIV and mpox virus had severe disease requiring hospitalisation. The administration of cidofovir was followed by rapid improvement (Fabrizio et al., 2022). All four patients on cidofovir recovered completely (Mondi et al., 2022).

Transient elevation in a hepatic enzyme was seen in one patient (Mondi et al., 2022). The other studies on cidofovir did not report any adverse events (Fabrizio et al., 2022; Mailhe et al., 2022; Moschese et al., 2022). The study employing topical cidofovir reported local adverse events, but there were not any systemic adverse events (Mondi et al., 2022; Sobral-Costas et al., 2022).

### 3.3 Brincidofovir (alternative name: CMX001, brand name: TEMBEXA^®^)

Brincidofovir (BCV) is a nucleotide analogue DNA polymerase inhibitor. It is a pro-drug composed of cidofovir conjugated to a lipid molecule. The lipid component resembles an endogenous lipid called lysophosphatidylcholine, allowing the molecule to enter the infected cells by taking on the natural lipid absorption mechanisms. Following absorption, the lipid molecule is broken down, releasing cidofovir for additional intracellular kinase phosphorylation to form cidofovir diphosphate, the active form of the drug. In contrast to cidofovir, brincidofovir does not act as a substrate for Organic Anion Transporter 1, which makes BCV less harmful to the kidneys. Therefore, brincidofovir has a higher safety profile for nephrotoxicity compared to cidofovir. Coming to preventive measures and adverse reactions of Brincidofovir, its administration requires continuous monitoring of hepatic function tests as it increases the serum transaminase and bilirubin levels. Other adverse effects seen are gastrointestinal side effects like diarrhea and vomiting. Pregnancy is ruled out before administering this drug as it is teratogenic in animal studies. Contraception is advised throughout the treatment and for 4 months after that. Brincidofovir is also known to have carcinogenic potential, so safety with handling is necessary. Brincidofovir is taken on an empty stomach or with a low-fat meal to increase the bioavailability of the drug. Drug-drug interactions are seen when used concomitantly with inhibitors of OATP1B1 and 1B3 (Organic Anion Transporting Polypeptide) like rifampin, erythromycin, and protease inhibitors like ritonavir as they increase its peak serum concentration, increasing the adverse events due to Brincidofovir (Das and Hong, 2019; National Center for Biotechnology Information, 2022).

Results with Brincidofovir have not been promising. In the solitary study on human patients, all three patients had to discontinue treatment because hepatic impairment led to hospitalization prolonging. Other issues encountered were conjunctivitis, lower limb abscess, and neuropsychiatric symptoms (Adler et al., 2022). They were hospitalized for 26–35 days. Thus, safety remains a key concern with this drug.

### 3.4 Vaccinia immune globulin (brand name: CNJ-016^®^)

Many medical countermeasures are kept on hand in case of orthopoxviruses as mpox emerges. Although most instances of mpox are minor and self-limited, supportive treatment is often enough to treat them. Most patients have moderate sickness and recover without medical help, but in very unwell or immunocompromised people, antivirals or vaccinia immune globulin (VIG) may be utilized. According to the Centre for Disease Control and Prevention (CDC), supportive care is often sufficient for people with a mpox virus infection because no particular medicines are available. Mpox virus disease can be prevented and treated similarly to other orthopoxvirus infections, and unless proven differently, all confirmed orthopoxvirus cases should be managed as though they are mpox (Centers for Disease Control and Prevention, 2022c; Rizk et al., 2022; UK Health Security Agency, 2022).

In immunosuppressed patients exposed to mpox for whom ACAM2000 vaccination is contraindicated, VIG, an injectable preparation of hyperimmune globulin made from the pooled blood of smallpox vaccine recipients, may be considered. These people’s acquired antibodies against the smallpox vaccine are removed and purified. Additionally, VIG can treat vaccinia virus-related aberrant infections brought on by autoinoculation, eczema vaccinatum, or severe generalized or progressive vaccinia (Hopkins and Lane, 2004; Weinstein et al., 2005). VIG is used to treat some vaccine-related side effects like infections due to the vaccinia virus in people with pre-existing skin disease and aberrant infections brought on by the vaccinia virus. VIG is not advised to treat post-vaccine encephalitis or encephalomyelitis, myopericarditis following smallpox vaccination, moderate cases of widespread vaccinia, erythema multiforme, or isolated vaccinia keratitis. Its use has not been evaluated in people with mpox or smallpox, even though it is a potential treatment. Data on its efficiency against these conditions are mostly sparse. Clinicians should use an Investigational New Drug (IND) application to administer VIG treatments. If tecovirimat is unavailable, the current Australian recommendations reserve VIG as a backup treatment for mpox infection (Australian Government Department of Health, 2022).

### 3.5 Trifluridine

Trifluridine is a fluorinated structural analogue of the DNA constituent thymidine. It acts by inhibiting DNA synthesis. It inhibits the enzymes involved in this process and may itself get incorporated into DNA. However, its action may lack selectivity. It has only been used as a topical preparation for the eye in cases of mpox virus infections. It is said to be safe when applied topically as eye drops. This is because it does not penetrate the intact cornea. However, in cases with corneal pathologies disrupting its structure, trifluridine may penetrate the cornea and be detectable in aqueous humor. Mild adverse events have been noted. These include transient local burning sensation, oedema of the eyelids, inflammation of the cornea, and allergy. It may be dosed at a 2-h interval till there is complete regeneration of epithelium in the cornea. Then, it may be administered once every 4 h for another 7 days. However, it is not prescribed for prolonged durations. In such cases where it has to be given beyond 3 weeks, alternative pharmacological options may be explored (Carmine et al., 1982).

Trifluridine has been used in mpox virus infection. However, its use in both the studies has been as an add-on agent. Overall, five patients received both Tecovirimat and local administration of Trifluridine. Though all these four patients varied greatly in their presentation, they were common in that all had ophthalmological manifestations of mpox virus disease too. Four of them recovered promptly and were discharged. One of them continues to develop worsening ocular symptoms and decreased visual acuity. No adverse events were reported with this drug (Cash-Goldwasser et al., 2022; Perzia et al., 2023).

### 3.6 New discoveries

Researchers are exploring several new potential drug targets and therapeutic options for the treatment of mpox amidst concerns regarding both supply shortages and drug resistance (Hudu et al., 2023; Rabaan et al., 2023). Mutations have been reported in both the F13L and D13L genes with potential for resistance (Garriga et al., 2018). This study has reported a frameshift mutation in the currently prevalent strain, sparking fears of drug resistance (Zhang et al., 2022). Thus, discovery of newer options is critical.

The genetic material of all orthopoxviruses show similarity. The open reading frames responsible for viral protein synthesis are well-conserved in orthopoxviruses. This is especially manifested in the case of the VP37 protein. Tecovirimat targets this protein and has shown positive results in mpox. Therefore, novel drugs can be developed focusing on the VP37 protein (Hudu et al., 2023). There are several other patents involving TPOXX. This includes US11433051B2 which comprises of simethicone and several other pharmaceutical excipients to enhance its action. US8642577B2 involves combining TPOXX with other antiviral drugs, and can be used for a wide range of orthopoxvirus diseases. Since TPOXX doesn’t inhibit viral nucleic acid synthesis, combining an envelope-formation inhibitor with DNA synthesis like Cidofovir targets mpox virus at two different levels can have synergistic action. CN115141136A proposes combining TPOXX with another crystalline ligand and can again be used for several orthopoxvirus infections. We have compiled the patents we feel are especially clinically relevant. A more comprehensive list can be found here (Almehmadi et al., 2022).

NIOCH-14 is another potential drug that has already cleared phase 1 clinical trial (Hudu et al., 2023). It is a prodrug of TPOXX, and is also administered as a capsule. Once inside the body, it quickly metabolises to TPOXX. It has shown similar or somewhat better efficacy and bioavailability compared to TPOXX in several *in-vitro* and *in-vivo* studies involving orthopoxviruses including mpox virus (Titova et al., 2015; Mazurkov et al., 2016; 2020; Sergeev et al., 2016). Thus, it is a promising medicine and the drug development process is expected to be complete by the next year (2024) (World Health Organization, 2022b).

Another drug inhibiting VP37 protein is N(1)-isonicotinoyl-N(2)-3-methyl-4-chlorobenzoylhydrazine. It has a similar mechanism of action, and inhibits envelopment, extracellular release and consequent propagation of disease in the human body. Though beneficial in *in-vitro* studies, the same results were not replicated in animal models (Prichard and Kern, 2005; Prichard and Kern, 2012). To add to all of this, there has been further exploration of the detailed structure of the VP37 protein. There has been greater insight into the allosteric site of the target protein, and how the inhibitor is dynamically flipped and it’s strong binding energy (Sen Gupta et al., 2023). This can lead to development of more optimally designed drugs acting on the VP37 protein.

Other potential drug targets could be E9L and A24R to arrest viral nucleic acid replication. These inhibit DNA polymerase and RNA polymerase respectively. While the former is required for mpox virus to replicate its own double stranded DNA, the latter is needed for protein synthesis. Drugs acting on A48R can act as nucleoside analogues to terminate chain prolongation. As discussed earlier, this is different to the case of Cidofovir. Though Cidofovir also acts as a nucleoside analogue, it inhibits human polymerases too (Chamberlain et al., 2019). Instead, a drug acting on A48R would inhibit phosphorylation of thymidine monophosphate which is structurally quite different from its human counterpart thereby imparting specificity of action. North-Methanocarbathymidine has also shown promising action (Smee et al., 2007). Aciclovir and KAY-2-41 were effective in *in-vitro* studies on orthopoxviruses (Sauerbrei et al., 2005; Duraffour et al., 2014). Nucleic acid replication can also be inhibited by targeting topoisomerases. Targeting H5R, B1R, and F10L can prevent phosphorylation thereby inhibiting tyrosine kinase. This approach has been successful previously in cytomegalovirus (Piret and Boivin, 2019). Similarly, the ErbB-1 kinase can be inhibited by epidermal growth factor signal transduction inhibitors. This again inhibits phosphorylation. Viral entry can be reduced by designing drugs acting on E8L and A6R. Interferons act on B8R and inhibit the terminal step of protein synthesis. Drugs acting on I7L and D13L can target preparation of viral core and membrane. Other drugs that can be used as a reference to design new drugs for mpox can be found here (Rabaan et al., 2023). Nanotechnology based drug administration and nanomedicines are also being explored (Dash and Kundu, 2023).

A detailed *in silico* study screened over 1000 approved drugs for activity against mpox viral proteins. Routinely used in oncology, fludarabine showed the best results with a high stability and docking score for A6R, a protein concerned with viral replication. On top of that, it also demonstrated activity against D8L involved in viral entry into human cells. Moreover, Fludarabine acts against F13L that codes for the VP 37 protein responsible for envelopment of mature virion particles as discussed earlier. Fludarabine also inhibits viral attachment to human cells by blocking an asparagine residue (Altayb, 2022). Fludarabine and its analogues can be studied *in-vitro* to generate more evidence regarding its activity in mpox. Norov-29 and Bemnifosbuvir have also demonstrated promising results with high binding free energies (Abduljalil and Elfiky, 2022). Another high throughput virtual screening has identified Naldemedine and Saquinavir to form stable complexes with mpox viral targets (Srivastava et al., 2023). A study has explored protein-protein interactions across the whole genome of several mpox strains and the human proteome. It identified several drugs including Fostamatinib and Tamoxifen (Kataria et al., 2023). This study identified 11 possible compounds for inhibition of thymidylate kinase, after screening hundreds of thousands of compounds (Sib Tul Hassan Shah and Naeem, 2023). Several small molecule inhibitors have also been studied shortlisting drugs like imatinib, conivaptan, lumacaftor, betulinic acid, and fluspirilene (Dutt et al., 2023; Khan et al., 2023).

Repurposing of drugs, and especially herbal formulations has long been seen as a practical solution in case of emerging and upcoming diseases with limited known therapeutic options. A study incorporating opinions of close to 300 herbal medicine practitioners found several formulations that are not studied enough for diseases like mpox. It includes Moringaceae, African palm oil, and Acacia pod extract. These can be studied for their antiviral efficacy, and depending on the results, taken up for further research (Abubakar et al., 2022). Several substances derived from curcumin have shown promising actions along with good pharmacokinetic properties and physiological stability (Akash et al., 2023). Traditional Chinese medicine is also being explored to find other options (Rong et al., 2023). There are several other studies focusing on repurposing of existing drugs for mpox (Arasu et al., 2023).

### 3.7 Vaccines

When it comes to the prevention of mpox virus infection, two vaccines are mainly considered. These are ACAM 2000, and JYNNEOS (also known as IMVANEX, IMAMUNE, and MVA). There are no approved vaccines specifically for mpox virus infection. The mpox virus and the variola virus (that causes smallpox) belong to the same genome of orthopoxvirus. Orthopoxviruses are known to share immunological cross-protection between them, but the evidence is not very strong. Immunity developed against smallpox may help protect against mpox virus infection too. Thus, smallpox vaccines are being repurposed for use in mpox virus infection (Huang et al., 2022; Akter et al., 2023).

JYNNEOS (or Imvamune) is a third-generation vaccine. It is modified vaccine Ankara (MVA), manufactured in Denmark. It comprises a virus that is incapable of replicating. It is approved in the United States for both smallpox and mpox virus infection. 0.5 mL of the vaccine is delivered subcutaneously 4 weeks apart. Then, the person is said to have been vaccinated 2 weeks after the second dose. It is also approved by the World Health Organization and medical agencies in Europe and Canada for post-exposure prophylaxis. There are several trials that are undergoing to test this vaccine further. Intradermal administration has also been practiced (Bloch et al., 2022; Huang et al., 2022; Jeyaraman et al., 2022). However, there are conflicting reports on its efficacy (Chakraborty et al., 2022). Breakthrough infection several weeks after vaccination has also been reported (Hazra et al., 2022).

ACAM 2000 is a second-generation vaccine. It is a derivative of the first-generation Dryvax vaccine (Nalca and Zumbrun, 2010). It differs from JYNNEOS in retaining the ability to replicate. Thus, it may cause severe adverse events like progressive vaccinia, eczema, cardiac injury and pericardial injury and can be unsafe in the immunocompromised population. Another disadvantage is that a bifurcated needle is used to puncture the skin at multiple places. Pustule formation at the site of vaccination indicates successful immune response and is labelled “take” of mpox vaccine. It is reserved for use in cases wherein JYNNEOS is contraindicated. It is only approved for smallpox and has not yet been approved for mpox virus infection. It has shown a protective action in monkey and dog models (Bloch et al., 2022; Huang et al., 2022; Jeyaraman et al., 2022). This has replaced the earlier Dryvax vaccine, which is the oldest smallpox vaccine globally (Chakraborty et al., 2022; Katamesh et al., 2023). LC16m8 and NYVAC are other third-generation vaccines. LC16m8 has considerably lesser replicative property, and is not given in immunosuppressed and those below 18 years of age. There have been several first-generation vaccines that are not in use currently. These are active replicating vaccines with varying reactogenicity. Some examples are Dryvax, Lister, EM-63, and Tian-Tian (Reina and Iglesias, 2023).

These vaccines have been associated with several adverse events including myocarditis and pericarditis (Food and Drug Administration, 2007; Voigt et al., 2016). Imvamune is considered unsafe for more than 15% of people living in the United States due to concerns over immunogenic adverse events (Rabaan et al., 2023). And it is considered safer than ACAM 2000. Both TPOXX and NIOCH-14 have been claimed to beneficial in avoiding vaccine side effects. However, drug-vaccine interactions should be carefully assessed and researched comprehensively before recommending it for routine use (Grosenbach et al., 2011).

Several other vaccines for mpox virus infection are under development or under study. Aventis Pastuer smallpox vaccine is being used under an investigational new drug protocol for smallpox. This may be later developed for use in mpox too (Rizk et al., 2022). A Japanese vaccine by the name of LC16m8 is of the replicating subtype and was used for smallpox. It has shown protective action against mpox virus infection in several animal models, including mice, rabbits, and non-human primates. A novel vaccine by the name of TNX-801 has been patented. It has also shown benefits in animal models like mice and macaques (Huang et al., 2022). mRNA vaccines with four to six antigens have been tested in mice. These have shown potent immune response (Zeng et al., 2023). Harnessing the potential of bioinformatics in designing new molecules for prevention of diseases including mpox virus infection, several studies have introduced designs for vaccines with multiple epitopes (Aiman et al., 2022; Akhtar et al., 2022; Aziz et al., 2022; Singh et al., 2023; Zaib et al., 2023). These researchers have developed two vaccine candidates targeting A35R, B6R, and H3L. Both the candidates have shown promising docking and dynamics for toll-like receptors 2 and 4, and major histocompatibility complexes (Tan et al., 2023). Another group developed two mRNA vaccine candidates with four to five components that have demonstrated immune response in mice (Zhang et al., 2023). These may be further tested along the process of drug development, like in the case of other novel molecules.

## 4 Discussion

This review elaborates on the different pharmacological treatment options for mpox virus infection. A bibliometric analysis was also carried out across several databases like PubMed, Scopus, and Embase. We observed that five drugs are mainly used for specific management of mpox disease in humans: tecovirimat, cidofovir, brincidofovir, trifluridine, and Vaccinia Immune Globulin. Tecovirimat has emerged as an exciting option with efficacy in progressive disease. No signals for safety concerns have been detected either. All other options are infrequently used. Cidofovir and its related compound brincidofovir are also used. The latter is linked with hepatic impairment, and treatment had to be discontinued in all three cases in a study. Vaccinia immune globulin has not been used much and is mainly preferred for other indications like post-vaccine complications. Trifluridine is successfully used as an add-on treatment option in patients with ocular manifestations of the mpox virus.

According to the interim guidelines by CDC for treating mpox virus infection, treatment should not be considered across all cases. It should only be considered based on clinical features and individual baseline risk. Clinically, severe disease and involvement of areas of the body that can potentially cause serious complications are conditions for treatment consideration. Pharyngeal lesions may lead to dysphagia and lack of control of secretions. Rectal involvement may lead to severe proctitis and pain. Treatment should also be considered in high-risk individuals like immunocompromised, pregnant, lactating, children, and those with dermatological diseases that affect cutaneous integrity (Centers for Disease Control and Prevention, 2022a; Gandhi et al., 2023; Rao et al., 2023).

These drugs still need to be adequately tested in well-designed studies on patients with monkeypox infection. The main reason behind the lack of such studies might be feasibility issues. Further studies, including randomized controlled trials like these (NCT05534984, NCT05534165), must confirm the results and optimize the dosage range.
